# The Effect of Positioning Guide Color on Shade Measurements Using a Clinical Spectrophotometer

**DOI:** 10.1590/0103-6440202405995

**Published:** 2024-10-28

**Authors:** Joao Arthur Dumke, Lauren Arrua Fantine, Suzana Nogueira, Bruna Gaidarji, Danielle Zorzo Righes, Bibiana Gabardo Perez Mariano da Rocha, Leticia Brandao Durand

**Affiliations:** 1 Post-Graduate Program in Oral Science, Division of Restorative Dentistry, Federal University of Santa Maria (UFSM).; 2 Post-Graduate Program in Clinical Dentistry, Federal University of Santa Catarina (UFSC).; 3 Private Practice.; 4 Post-Graduate Program in Oral Science, Division of Restorative Dentistry, Federal University of Santa Maria (UFSM), Atitus Educação.; 5 Post-Graduate Program in Oral Science, Division of Restorative Dentistry, Federal University of Santa Maria (UFSM).; 6 Department of Restorative Dentistry, Federal University of Santa Maria (UFSM), Santa Maria, Rio Grande do Sul, Brazil.; 7 Department of Restorative Dentistry, Federal University of Santa Maria (UFSM), Santa Maria, Rio Grande do Sul, Brazil.

**Keywords:** Differential threshold, color perception, colorimetry, dental materials, dental research

## Abstract

The purpose of this study was to investigate the influence of the color of positioning guides on the CIEL*a*b* color coordinates of ceramic, resin composite and bovine tooth surfaces measured by a clinical spectrophotometer. Positioning guides (n=10) in different colors (translucent, purple, yellow, green and, blue) were made for each surface (ceramic, resin composite and bovine tooth). The CIEL*a*b* color coordinates were measured with the positioning guides and with no positioning guide (control group). The ΔL*, Δa*, Δb*, ΔE*_ab_, and ΔE_00_ were calculated between the control group and the different groups. The CIEL*a*b* color coordinates were analyzed by one-way ANOVA followed by the post-hoc Tukey test (α=0.05). Color differences were interpreted using the 50:50% perceptibility and acceptability visual thresholds. Positioning guides with different colors presented significant differences for all surfaces and color coordinates. ΔE*_ab_, and ΔE_00_ mean values for all surfaces and positioning guide colors exceeded the acceptability visual thresholds, except for the bovine tooth surface measured with the green positioning guide. The shade measurement was affected by the color of the positioning guides regardless of the surface that was evaluated.

## Resumo

O objetivo deste estudo foi investigar a influência da cor do guia de posicionamento nas coordenadas de cor CIEL*a*b* de superfícies de cerâmica, resina composta e dentes bovinos medidas por espectrofotômetro clínico. Guias de posicionamento (n=10) com cores diferentes (translúcida, roxo, amarelo, verde e azul) foram confeccionadas para cada superfície (cerâmica, resina composta e dente bovino). As coordenadas de cor CIEL*a*b* foram medidas com as guias de posicionamento e sem guia de posicionamento (grupo controle). Os ΔL*, Δa*, Δb*, ΔE*_ab_ e ΔE_00_ foram calculados entre o grupo controle e os diferentes grupos. As coordenadas de cor CIEL*a*b* foram analisadas por ANOVA unidirecional seguida pelo teste post-hoc de Tukey (α=0,05). As diferenças de cor foram interpretadas utilizando os limiares visuais de perceptibilidade e aceitabilidade de 50:50%. Guias de posicionamento com cores diferentes apresentaram diferenças significativas para todas as superfícies e coordenadas de cor. Os valores médios de ΔE*_ab_ e ΔE_00_ para todas as superfícies e cores do guia de posicionamento excederam os limites de aceitabilidade visual, exceto para a superfície dentária bovina medida com o guia de posicionamento verde. A aferição da cor foi afetada pela cor das guias de posicionamento independentemente da superfície avaliada.

## Introduction

The accuracy and precision of dental shade selection present major challenges for dental technicians and clinicians, and depend on several factors, such as the characteristics of the shade guides, the color matching ability of the observer, and the lighting conditions ^1^. Communication between professionals is often conducted using shade guides ^2^ ^,^ ^3^, which are widely used in dentistry, and considered as the most common method for shade selection ^3^. The main advantages of this system is that it is affordable and practicable, however, shade guides may undergo color changes over the years due to the disinfection processes, sterilization cycles, and aging ^4^. Moreover, this method is limited due to its subjective nature and its inability to cover all the ranges of dental shades. Furthermore, color discrepancy among different shade guides from the same manufacturer has been reported ^4^ ^,^ ^5^ ^,^ ^6^.

Successful shade selection, adequate color communication, and color reproduction can be achieved by using clinical spectrophotometers ^7^. This equipment is able to determine the color by assessing the spectral reflectance or transmittance curve of objects, and provides the measurement of the color coordinates of the CIEL*a*b* color system ^8^. Thus, reducing the subjectivity of the visual color assessment and shade selection. However, apart from the expenses regarding equipment acquisition, training is required for obtaining accurate color measurements ^3^ ^,^ ^9^.

During visual color assessment, teeth must be clean and kept moist to avoid dehydration. The environment should preferably have natural lighting, and the indoor walls should be neutral in colors. The examiner should be positioned at the patient's eye level, and the shade selection process should be quick to prevent eye fatigue ^4^. Additionally, it is essential to position the shade tab near the tooth, ensuring incisal alignment between the shade tab and the tooth ^4^ ^,^ ^10^. Conversely, when carrying out instrumental color assessment, the dentist should perform three measurements at the same dental location to guarantee color reproducibility. Additionally, the clinical spectrophotometer must be calibrated before each color assessment ^11^ ^,^ ^12^.

When color assessment is conducted in clinical and laboratory research, the area of the instrumental color assessment must be systematically performed at the same exact location to obtain precise readings ^13^. The use of a positioning guide is necessary in order to guarantee proper placement of the clinical spectrophotometer tip and for standardization purposes ^14^. The positioning guide improves the accuracy, precision, and reproducibility of the color measurements ^13^. In previous in vitro studies, the utilization of positioning guides has been reported ^15^ ^,^ ^16^. Additionally, clinical studies have also recommended their use ^17^ ^,^ ^18^. However, depending on the material used to produce the positioning guides, they may exhibit different colors ^15^ ^,^ ^17^ ^,^ ^19^ ^,^ ^20^.

A clinical trial demonstrated the influence of the color of positioning guides on the measurement of tooth whitening outcomes ^17^. It is important to note that this study was not originally designed to examine the color of the positioning guides, analyzing only two different colors, exclusively on the enamel surface ^17^. Recently, a clinical study conducted by the same research group recommended the use of bleaching trays made of ethylene vinyl acetate as repositioning guides or the freehand method when assessing color of dental surfaces ^18^. However, at present, the authors are unaware of any recommendation regarding which material or color should be used for the fabrication of the positioning guides when measuring resin composite and ceramic surfaces. Information concerning the influence of the color of the positioning guide on the CIEL*a*b* color coordinate measurements for different surfaces are necessary for both clinical and laboratory research. In this context, the aim of the present study was to investigate the influence of the color of the positioning guide on the CIEL*a*b* color coordinates of ceramic, resin composite and bovine tooth surfaces, measured by a dental spectrophotometer. The tested hypothesis considers that the color of the positioning guides influences the CIEL*a*b* color coordinates of ceramic, resin composite and bovine tooth surfaces leading to color differences exceeding the perceptibility and acceptability visual thresholds for color discrimination.

## Material and Methods

### Study Design

The present study was designed to evaluate the influence of the color of the positioning guide on the clinical spectrophotometer measurement of the CIEL*a*b* color coordinates of ceramic, resin composite and bovine tooth surfaces. The sample size was estimated based on a previous pilot study using an online sample calculator - http://www.estatistica.bauru.usp.br. Sample size was calculated for each CIEL*a*b* color coordinate considering six ^6^ independent variables (control, translucent guide, purple guide, yellow guide, green guide and blue guide). The standard deviation used was 0.22, 0.17 and 0.52 for L*, a* and b*, respectively. The minimum detectable difference used was based on the 50:50% acceptability visual threshold for ΔL*=1.25, Δa*=1.25 and Δb*=2.80, previously reported ^21^. A significance level of 5% (alpha) and a power of 80% (1-beta) was set. In addition, previous in vitro studies have used sample sizes ranging from 5 to 10 specimens. Based on sample sizes from previously published studies using either extracted bovine or human teeth, a sample size of n=10 was adopted ^16^ ^,^ ^22^ ^,^ ^23^ ^,^ ^24^ ^,^ ^25^.

### Surface Preparation

Three different surfaces were prepared for this study: ceramic, resin composite and bovine tooth. For the ceramic surface a ceramic disc with 11-mm diameter and 2-mm thickness was fabricated from A2 opaque shade feldspathic ceramic (VM13, Vita Zahnfabrik, Bad Säckingen, Germany). A thickness of 2 mm was required in order to ensure an optical behavior comparable to of an opaque body, without the interference of the background color ^26^.

A resin composite disc was produced from A2 dentin shade Filtek Z350 XT resin composite (3M ESPE, St. Paul, MN, USA) with 11 mm diameter and 4 mm thickness. The resin composite was inserted into a metallic device in a single increment and light-cured for 40 seconds on both sides using a light-emitting-diode (LED; Bluephase, Ivoclar Vivadent, Schaan, Liechtenstein) with 1000 mW/cm^2^ irradiance. For the resin composite surface, a 4 mm thickness was required to be considered the inherent color of the resin composite which is not affected by the background color ^27^.

For the bovine tooth surface, a bovine tooth was selected, and the buccal surface of the tooth was flattened with a sandpaper sheet with 400 grit for 60 seconds under running water.

### Positioning guide

**Figure 1:**
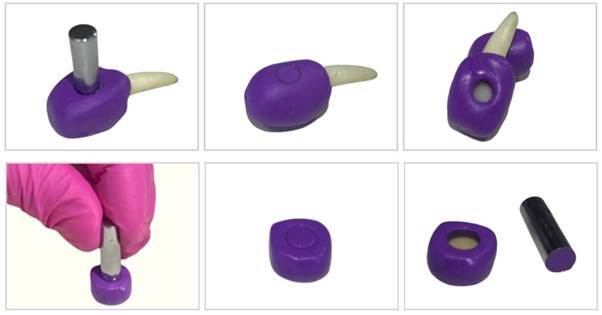
Fabrication process and the format of the positioning guides

The positioning guides for each group and surface (n=10) were prepared in different colors: translucent (addition silicone), purple (addition silicone), yellow (addition silicone), green (condensation silicone), and blue (polyether). The materials were manipulated and prepared individually according to the manufactures’ instructions. After complete polymerization of the material, a 6 mm diameter cylindrical metallic device was used to obtain a standardized central window, that was equivalent to the diameter of the spectrophotometer tip standardizing the exact location for the color measurement of each surface. All positioning guides were 3.0 mm thick and had the following CIEL*a*b* color coordinates: Purple (L* = 34.17, a* = 41.50, b* = -48.56), Yellow (L* = 67.57, a* = 7.25, b* = 69.28), Green (L* = 47.51, a* = -21.76, b* = 1.70), and Blue (L* = 43.89, a* = 3.39, b* = -25.80). The material, the manufacturer, the color, the batch number, and the composition of each material used for the fabrication of the positioning guides are presented in Box 1. Figure 1 illustrates the fabrication process and the format of the positioning guides.

**Box 1:**
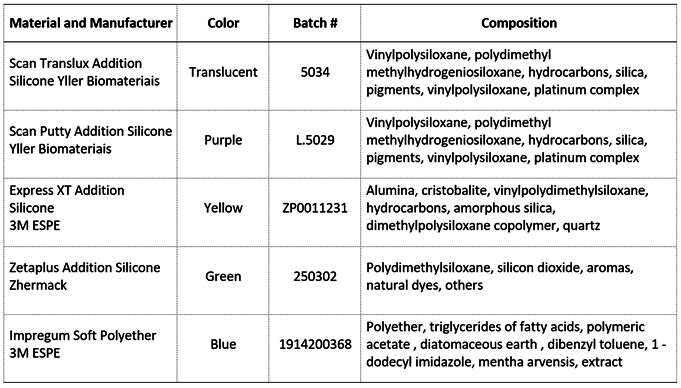
Composition and information regarding the materials used to produce the positioning guides

### Instrumental color assessment

The color of each surface was measured using the Vita Easyshade Advanced 4.0 spectrophotometer (VITA-Zahnfabrik, Bad Säckingen, Germany). Each measurement was performed in triplicate by the same operator, with similar conditions of illumination and temperature. The color measurement process was standardized by consistently conducting it in the same room, maintaining a stable environment with the air conditioning set to an average temperature of 21 degrees Celsius. Additionally, measurements were consistently taken at the same time of day to ensure uniform lighting conditions throughout the study. The clinical spectrophotometer was systematically calibrated before the instrumental color assessments. CIEL*a*b* color measurements of each surface without the use of any positioning guide were considered as the control group. The color difference was calculated between the control group (without positioning guide) of the tested surfaces (dental, ceramic and resin composite) and each positioning guide group (translucent, yellow, purple, green and blue) as follows:

$${\Delta E}_{ab}^{*}=\sqrt{{\left(\Delta L^{*}\right)}^{2}+{\left(\Delta a^{*}\right)}^{2}+{\left(\Delta b^{*}\right)}^{2}}$$

$${∆E}_{00}={\left[{\left(\frac{∆L^{'}}{k_{L}S_{L}}\right)}^{2}+{\left(\frac{∆C^{'}}{k_{C}S_{C}}\right)}^{2}+{\left(\frac{∆H^{'}}{k_{H}S_{H}}\right)}^{2}+R_{T}\left(\frac{∆C^{'}}{k_{C}S_{C}}\right)\left(\frac{∆H^{'}}{k_{H}S_{H}}\right)\right]}^{\frac{1}{2}}$$

### Statistical analysis

The CIEL*a*b* color coordinates were analyzed by the one-way ANOVA followed by the post-hoc Tukey test (α=0.05) using a standard statistical software package SPSS Statistics 20.0 (IBM Armonk, New York, USA). 

### Interpretation of the color differences

The CIEDE2000 and CIEL*a*b* color differences were interpreted using the 50:50% perceptibility (PT) and acceptability (AT) visual thresholds: PT=1.2 ΔE*_ab_ units, AT=2.7 ΔE*_ab_ units, PT_00_=0.8 ΔE_00_ units and AT_00_=1.8 ΔE_00_ units ^28^ ^,^ ^29^. The mean values of ΔL*, Δa*, Δb* were interpreted based on 50:50% PT and AT for ΔL*=1.25, Δa*=1.25, and Δb*=2.8 ^21^.

## Results

The comparisons of the CIEL*a*b* color coordinates among the control group and each positioning guide for ceramic, resin composite and bovine tooth surfaces are presented in Table 1. The clinical spectrophotometer measurement of the CIEL*a*b* color coordinates presented statistically significant differences for L* (P< 0.001), a* (P< 0.001) and b* (P< 0.001) color coordinates for all tested surfaces. 

All positioning guide groups presented significant differences in comparison to the control group for the L* color coordinate for the ceramic surface. As for the resin composite surface, only the yellow positioning guide group showed mean values of L* statistically different from the control group. In the bovine tooth surface, only the green positioning guide group presented mean values of L* similar the control group.

All positioning guide groups presented significant differences in comparison to the control group for the a* color coordinate for all tested surfaces. For the b* coordinate, all positioning guide groups presented significant differences in comparison to the control group for both ceramic and bovine tooth surfaces. However, for the resin composite surface, the translucent, purple and blue positioning guide groups obtained mean values statistically similar the control group.

**Table 1:** Mean and standard deviation values of the CIEL*a*b* color coordinates for ceramic, resin composite and bovine tooth surfaces

Guide	L*	a*	b*
Ceramic			
Control	88.34 (0.05)^A^	1.28 (0.04)^C^	30.50 (0.1)^B^
Translucent	85.14 (0.05)^D^	-0.27 (0.06)^E^	25.60 (0.20)^DE^
Purple	85.44 (0.03)^D^	1.53 (0.01)^B^	25.11 (0.12)^E^
Yellow	87.27 (0.42)^B^	4.21 (0.15)^A^	31.47 (0.79)^A^
Green	85.87 (0.08)^C^	-1.50 (0.01)^F^	26.53 (0.03)^CD^
Blue	84.54 (0.28)^E^	0.44 (0.04)^D^	26.90 (0.88)^C^
Resin Composite			
Control	77.88 (0.04)^B^	7.12 (0.13)^A^	39.42 (0.19)^C^
Translucent	79.79 (0.10)^B^	3.05 (0.11)^D^	39.93 (0.62)^C^
Purple	79.70 (0.23)^B^	4.27 (0.31)^B^	39.85 (0.31)^C^
Yellow	81.43 (0.29)^A^	3.51 (0.25)^C^	44.49 (0.59)^A^
Green	79.56 (0.03)^B^	3.51 (0.11)^C^	41.14 (0.29)^B^
Blue	78.17 (0.16)^B^	3.23 (0.08)^CD^	39.77 (0.50)^C^
Bovine Tooth			
Control	86.44 (0.22)^B^	0.72 (0.08)^D^	32.44 (0.21)^C^
Translucent	90.24 (0.13)^A^	-1.22 (0.19)^E^	21.03 (1.36)^D^
Purple	84.37 (0.99)^C^	3.27 (0.23)^B^	38.33 (1.27)^AB^
Yellow	84.62 (0.58)^C^	3.92 (0.13)^A^	38.09 (1.00)^A^
Green	86.93 (0.17)^B^	1.42 (0.07)^C^	35.68 (0.18)^B^
Blue	82.12 (0.54)^D^	2.94 (0.28)^B^	37.20 (0.98)^AB^

* Different uppercase letters in the same column indicate statistically significant differences (p>.05). Standard deviation values are in parentheses

The mean ΔL*, Δa*, Δb*, ΔE*_ab_ e ΔE_00_ values and standard deviations for the ceramic, resin composite and bovine tooth surfaces and the AT visual thresholds are presented in Table 2. Considering the ceramic surface, all mean ΔL* values were above AT except the yellow positioning guide group. For Δa*, only the purple and blue positioning guide groups presented mean values within the limits of AT. As for the mean Δb* values for the ceramic surface, all groups except the yellow positioning guide group presented mean values above the AT and all groups presented mean ΔE*_ab_ e ΔE_00_ exceeding the AT. 

For the resin composite surface, all positioning guide groups presented mean ΔL* values above the AT, except for the blue positioning guide group. Additionally, only the yellow positioning guide group presented mean Δb* values within the limits of AT. Moreover, all positioning guides presented mean Δa*, ΔE*_ab_ e ΔE_00_ values above the AT for the resin composite surface.

For the bovine tooth surface, only the green positioning guide group presented mean ΔL*, Δa* e ΔE_00_ within the limits of AT, however, for Δb* and ΔE*_ab_ all positioning guide groups presented mean values above AT.

**Table 2:** Mean and standard deviation values for ΔL*, Δa*, Δb*, ΔE*_ab_ e ΔE_00_ of ceramic, resin composite and bovine tooth surfaces

Guide	ΔL*	Δa*	Δb*	ΔE*_ab_	ΔE_00_
Ceramic					
Translucent	3.20 (0.09)	1.68 (0.04)	4.90 (0.14)	6.05 (0.09)	3.26 (0.04)
Purple	2.90 (0.07)	0.27 (0.06)	5.39 (0.17)	6.12 (0.12)	3.06 (0.04)
Yellow	1.07 (0.40)	3.07 (0.19)	0.97 (0.81)	3.36 (0.31)	2.51 (0.16)
Green	2.47 (0.09)	2.99 (0.05)	3.97 (0.10)	5.44 (0.11)	3.35 (0.06)
Blue	3.80 (0.32)	0.90 (0.01)	3.60 (0.80)	5.33 (0.60)	3.01 (0.25)
Resin Composite					
Translucent	1.91(0.09)	4.10 (0.21)	0,51 (0.44)	4.54 (0.22)	3.34 (0.20)
Purple	1.82 (0.26)	2.88 (0.40)	0.43 (0.52)	3.41 (0.48)	2.50 (0.36)
Yellow	3.55 (0.25)	3.63 (0.29)	5.07 (0.73)	7.19 (0.43)	4.27 (0.09)
Green	1.68 (0.22)	3.64 (0.21)	1.72 (0.70)	4.35 (0.15)	3.10 (0.12)
Blue	0.29 (0.21)	3.93 (0.15)	0.35 (0.55)	3.95 (0.21)	2.93 (0.15)
Bovine Tooth					
Translucent	3.80 (0.21)	2.15 (0.30)	11.41 (1.53)	12.19 (1.46)	6.01 (0.74)
Purple	1.96 (1.10)	2.61 (0.24)	5.04 (2.65)	6.08 (2.47)	3.04 (0.49)
Yellow	1.82 (0.62)	3.26 (0.19)	5.65 (2.31)	6.77 (1.13)	3.39 (0.43)
Green	0.49 (0.25)	0.71 (0.08)	3.24 (0.32)	3.35 (0.14)	1.41 (0.05)
Blue	4.32 (0.51)	2.27 (0.32)	4.76 (1.72)	6.83 (0.70)	3.77 (0.32)

** Bold numbers located in the ΔE_00_ and ΔE*_ab_ column indicate values below the 50:50% AT threshold (ΔE_00_=1.8 and ΔE_ab_=2.7);^28^ ^,^ ^29^ Bold numbers located in the ΔL*, Δa*, Δb* column indicate values below the 50:50% AT and PT thresholds 1.25, 1.25 and 2.8, respectively.^21^

## Discussion

The present study evaluated the influence of the color of the positioning guide on dental spectrophotometer measurements of CIEL*a*b* color coordinates for ceramic, resin composite and bovine tooth surfaces. The use of positioning guides to standardize the location of instrumental color assessment in clinical and laboratory studies is a recommended practice ^13^ ^,^ ^17^ ^,^ ^18^ ^,^ ^30^. Although recent research has explored the influence of positioning guide color on instrumental color assessments ^17^ ^,^ ^18^, more data are needed on how the color and material of these guides affect the CIELab* color coordinates of dental, ceramic, and resin composite surfaces, measured by clinical spectrophotometers.

Research has been conducted using positioning guides produced from custom-made trays of ethylene-vinyl acetate (EVA) ^13^ ^,^ ^31^, while other investigations used positioning guides made from materials with different colors ^16^ ^,^ ^17^ ^,^ ^30^. This study evaluated the instrumental color assessment of three different surfaces with translucent (additional silicone), purple (additional silicone), yellow (additional silicone), green (condensation silicone), and blue (polyether) positioning guides in order to assess a range of possible color options available for the fabrication of positioning guides. CIEL*a*b* color coordinates measured from each surface without the use of any positioning guide was used as the control group considering that the manufacturer of the clinical spectrophotometer does not indicate the use of positioning guides during instrumental color assessment, and due to the fact that the CIEL*a*b* color coordinates are measured by the clinical spectrophotometer through light reflection ^32^.

The tested hypothesis was accepted because the use of positioning guides with different colors affected the CIEL*a*b* color coordinates measured by the clinical spectrophotometer for all tested surfaces (ceramic, resin composite and bovine tooth) generating, in most groups and surfaces, mean values of color differences (ΔL*, Δa*, Δb*_,_ ΔE*_ab_ and ΔE_00_) above the 50:50% acceptability visual thresholds ^21^ ^,^ ^28^ ^,^ ^29^.

The use of visual thresholds is important because it provides clinical relevance to instrumental data, indicating what is visually perceived or not, and interpreting numerical values into what may be clinically distinguished ^33^. The 50:50% PT and AT visual thresholds used in the present study to interpret the CIEL*a*b* and CIEDE2000 color differences are standardized and recommended by ISO for color compatibility studies in dentistry ^28^ ^,^ ^29^ ^,^ ^33^. Additionally, the 50:50% AT and PT visual thresholds for of ΔL*, Δa* and Δb* ^(^ ^21^ were also used for the interpretation providing clinical significance to the brightness (L*) and color differences in the red-green (a*) and blue-yellow (b*) axes. 

In addition to examining whether there is a color difference between surfaces tested with and without positioning guides, it is crucial to identify the extent to which each color coordinate (L*, a*, b*) is influenced by the positioning guide. It is also essential to determine the directions in which the L*, a*, and b* color coordinates shift for each positioning guide and surface tested. 

In general, on the ceramic surface, a decrease in lightness compared to the control group was observed for all positioning guides. Regarding the a* coordinate, the translucent, green, and blue guides showed a decrease in a*, indicating a shift towards green, while the yellow and purple guides showed an increase in a*, indicating a shift towards red compared to the control group. For all positioning guides, except the yellow one, the b* coordinate presented a reduction in the mean values, indicating a shift towards blue compared to the control group. Conversely, for the resin composite surface, an increase in lightness, a reduction in a*, and an increase in b* was observed compared to the control group for most of the positioning guide colors. For the dental surface, a decrease in L*, an increase in a*, and an increase in b* for all positioning guides, except the translucent guide, were observed in comparison to the control group.

In practical terms, this means that dental surfaces, measured with positioning guides of different colors, may tend to present less luminous, more reddish, and more yellowish results when compared to the control group. The resin composite surface may show more luminous, more yellowish, and more greenish results, regardless of the color of the positioning guide. However, ceramic surfaces may present less luminous results when color is measured with positioning guides, and the direction of the a* and b* color coordinates shift may vary depending on the color of the positioning guide. However, when a translucent positioning guide was used on the bovine dental surface an increase in L* and a decrease in the a* and b* color coordinates was observed. This particular result aligns with a clinical study conducted by Santana et al ^18^, which found that the translucent guide produced readings with higher L* and lower a* values for central incisors. 

The use of positioning guides is particularly recommended for laboratory and clinical studies investigating optical properties, as it ensures consistency and precision in color measurements. The present study assessed the color of a bovine tooth surface. This decision was considered because in dental in vitro research, bovine teeth can effectively substitute human teeth due to their similar enamel microstructure ^34^. Bovine teeth were chosen because they are easier to obtain compared to maxillary human incisors. Furthermore, the larger dimensions and simplified anatomical features of bovine teeth facilitated the creation of a flat buccal surface and the confection of the positioning guides.

The findings of the present study are important and should be taken into account when using positioning guides, given the expanded use of clinical spectrophotometers as an efficient and reliable source of data collection and communication for research and clinical application ^9^ ^,^ ^16^ ^,^ ^20^. Moreover, the spectrophotometer is widely used in different fields. In research it can be applied to observe immediate and long term effects of bleaching treatments ^19^ ^,^ ^31^, to investigate the masking of discolored substrates ^35^, to evaluate color stability of restorative materials ^3^ ^,^ ^20^, and a variety of other research possibilities. Clinically, it is widely used as an objective tool for shade selection and communication between professionals and dental technicians, being more accurate than visual assessments ^2^ ^,^ ^3^. Understanding the impact of the use of positioning guides on color measurement is important because it may influence the results of clinical and laboratory studies. This information is particularly relevant because, according the results of our study, in most cases, color changes may be visually perceived. The overall deltas for each of the evaluated variables presented most of the mean values (84%) above the 50:50% AT. This result demonstrates that, beyond numerical and statistical differences, the color changes can be visually identified, with values exceeding the threshold of acceptability.

Future studies are recommended to address this aspect clinically and to evaluate the influence of different backgrounds and substrates. However, although the present study did not consider these variables, the results demonstrated that the color of the positioning guide should be taken into consideration when using a clinical spectrophotometer, regardless of whether the surface is dental, ceramic, or resin composite. 

Spectrophotometers determine tooth color by measuring the amount and spectral composition of the reflected light of an object, taking into account the different curvature axes ^15^. Thus, the color of the background could have influenced the instrumental color assessments because the positioning guides covered both the top and bottom surfaces of the resin composite and ceramic discs, as well as the buccal and palatal surfaces of the bovine tooth. Additionally, the reflection of the color of the positioning guide could have influenced the results ^18^. To eliminate any potential influence of background color on color measurement, the ceramic and resin composite surfaces were fabricated with an A2 opaque shade and thicknesses of 2 mm and 4 mm, respectively. These dimensions, supported by existing literature ^26^ ^,^ ^27^, effectively prevent background color from affecting color measurement.

Although benchtop spectrophotometers present more accurate results, dental spectrophotometers are essential for measuring color in clinical practice, and for curved surfaces such as teeth and restorations ^7^ ^,^ ^15^. The device used in the present study was previously calibrated before each color measurement and presents excellent accuracy and repeatability, being a precise and reliable tool for color determination ^7^. The present study did not evaluate the repeatability of the color assessments, however, a recent study ^18^ found low data variability in repeated measurements with a clinical spectrophotometer. Additionally, research has demonstrated that the Vita Easyshade exhibits excellent accuracy and repeatability, providing further support for its use in both research and clinical applications ^15^ ^,^ ^36^.

One limitation of the current study is that Vita Easy Shade is primarily intended for evaluating dental and ceramic surfaces. As a result, it may not be the most suitable tool for determining the color of composite specimens. However, the focus of this study was to determine whether the color of the positioning guide would affect the color measurement of different surfaces. By concentrating on the impact of the positioning guide color, this study provided insights that could be relevant across various materials and research applications.

The present study addresses answers to questions raised by two recently published articles that investigated the impact of the positioning guide colors on instrumental color assessments ^17^ ^,^ ^18^. Apart from the positioning guides with different colors, the present study tested a translucent positioning guide (Scan Translux addition silicone), with the expectation of obtaining results with less impact on the color coordinates measured by the clinical spectrophotometer. Nevertheless, contrary to what was expected, the translucent positioning guide also showed mean values exceeding the limits of AT for the ceramic surface and bovine tooth. Similarly, for the resin composite surface, the use of the translucent guide generated results below the AT only for Δb*. This phenomenon can be attributed to the thickness of the translucent guides, as their opacity tends to increase with greater thickness ^18^. Thus, transparent positioning guides such as those produced for custom bleaching trays may reduce the impact of the positioning guide on instrumental color assessment. However, Santana et al. (2024) recommends freehand color measurement due to the high reproducibility of the Easy Shade ^18^. In addition, this recommendation is supported by their findings, which indicate that, even extremely thin and translucent guides, such as those made from bleaching trays, demonstrated color differences above the PT and AT thresholds for central incisors ^18^. 

The present in vitro experiment examined three distinct surfaces - resin composite, ceramic, and bovine tooth - it is suggested that other restorative materials, substrates and dental structures to be evaluated in both laboratory and clinical settings. More translucent materials, with greater light penetration, may possibly present different results. In addition, further research should be conducted to better understand the influence of positioning guides on color measurement by clinical spectrophotometers. Most importantly, attention is needed when selecting the materials used for positioning guides, as well as their respective colors, since this decision may affect the color coordinates measured by clinical spectrophotometers and consequently influence outcomes of investigations, regardless of whether the surface is dental, ceramic, or resin composite. Based on the findings of this in vitro study, the CIEL*a*b* measurements were influenced by the color of the positioning guides. The CIEL*a*b* and CIEDE2000 color differences were greater than the 50:50% PT for all positioning guides and surfaces tested. 
